# Retraction Note: Oxidized Low-density Lipoprotein (ox-LDL) Cholesterol Induces the Expression of miRNA-223 and L-type Calcium Channel Protein in Atrial Fibrillation

**DOI:** 10.1038/s41598-018-32554-4

**Published:** 2018-10-30

**Authors:** Fengping He, Xin Xu, Shuguo Yuan, Liangqiu Tan, Lingjun Gao, Shaochun Ma, Shebin Zhang, Zhanzhong Ma, Wei Jiang, Fenglian Liu, Baofeng Chen, Beibei Zhang, Jungang Pang, Xiuyan Huang, Jiaqiang Weng

**Affiliations:** Department Institute of Cardiovascular Diseases, The Yuebei People’s Hospital, Medical College, Shantou University, Shantou, Guangdong, China

Retraction of: *Scientific Reports* 10.1038/srep30368, published online 04 August 2016

This article is being retracted following an internal investigation which confirmed duplication of western blot data in Figure 4 and Supplementary Figure 4 and a level of image processing that is not compliant with the journal’s policies on image data integrity. The authors were unable to provide original data that matched the data in the published figures.

Fengping He, Xin Xu and Jiaqiang Weng agreed to the retraction. The other authors could not be reached.

